# Molecular Diagnosis of Neonatal Diabetes Mellitus Using Next-Generation Sequencing of the Whole Exome

**DOI:** 10.1371/journal.pone.0013630

**Published:** 2010-10-26

**Authors:** Amélie Bonnefond, Emmanuelle Durand, Olivier Sand, Franck De Graeve, Sophie Gallina, Kanetee Busiah, Stéphane Lobbens, Albane Simon, Christine Bellanné-Chantelot, Louis Létourneau, Raphael Scharfmann, Jérôme Delplanque, Robert Sladek, Michel Polak, Martine Vaxillaire, Philippe Froguel

**Affiliations:** 1 CNRS-UMR-8199, Univ Lille Nord de France, UDSL, Lille, France; 2 Inserm-U845, Department of Pediatric Endocrinology, Necker Enfants Malades Hospital, Université Paris Descartes, Paris, France; 3 Department of Genetics, Pitié-Salpétrière Hospital, Paris, France; 4 Department of Human Genetics, Faculty of Medicine, McGill University, Montreal, and Genome Quebec Innovation Centre, Montreal, Canada; 5 Department of Genomics of Common Disease, School of Public Health, Imperial College London, Hammersmith Hospital, London, United Kingdom; Peninsula Medical School, United Kingdom

## Abstract

**Background:**

Accurate molecular diagnosis of monogenic non-autoimmune neonatal diabetes mellitus (NDM) is critical for patient care, as patients carrying a mutation in *KCNJ11* or *ABCC8* can be treated by oral sulfonylurea drugs instead of insulin therapy. This diagnosis is currently based on Sanger sequencing of at least 42 PCR fragments from the *KCNJ11*, *ABCC8*, and *INS* genes. Here, we assessed the feasibility of using the next-generation whole exome sequencing (WES) for the NDM molecular diagnosis.

**Methodology/Principal Findings:**

We carried out WES for a patient presenting with permanent NDM, for whom mutations in *KCNJ11*, *ABCC8* and *INS* and abnormalities in chromosome 6q24 had been previously excluded. A solution hybridization selection was performed to generate WES in 76 bp paired-end reads, by using two channels of the sequencing instrument. WES quality was assessed using a high-resolution oligonucleotide whole-genome genotyping array. From our WES with high-quality reads, we identified a novel non-synonymous mutation in *ABCC8* (c.1455G>C/p.Q485H), despite a previous negative sequencing of this gene. This mutation, confirmed by Sanger sequencing, was not present in 348 controls and in the patient's mother, father and young brother, all of whom are normoglycemic.

**Conclusions/Significance:**

WES identified a novel *de novo ABCC8* mutation in a NDM patient. Compared to the current Sanger protocol, WES is a comprehensive, cost-efficient and rapid method to identify mutations in NDM patients. We suggest WES as a near future tool of choice for further molecular diagnosis of NDM cases, negative for chr6q24, *KCNJ11* and *INS* abnormalities.

## Introduction

Neonatal diabetes mellitus (NDM) is a rare monogenic form of non-autoimmune diabetes which affects 1 in ∼300,000 live births and is diagnosed before six months of age [1], [2], [3]. Approximately half of the NDM cases are transient (TNDM) but can ultimately relapse. In contrast, permanent NDM (PNDM) cases need continual treatment from diagnosis [1], [2], [3]. More than half of both forms of NDM cases have been elucidated, so far, and it appears that the genetic aetiologies of NDM are quite heterogeneous. Indeed, although the majority of TNDM cases have an abnormality in chromosome 6q24 and the other most frequent causes of NDM are missense mutations in the pancreatic β-cell K_ATP_ channel genes *KCNJ11* and *ABCC8*, and in the preproinsulin gene, NDM has been linked to numerous other genetic causes including point mutations in *GCK*, *GLIS3*, *EIF2AK3*, *PDX1*, *PTF1A*, *SLC2A2, HNF1B* or *FOXP3* [1], [2], [3].

Even if the presence of specific clinical features (*e.g.* relatively late age of onset, pancreas agenesis, developmental delay, renal failure, anaemia, thyroid disease, cardiac disorders…) or a family history of diabetes or consanguinity may suggest potential molecular aetiology(ies) for NDM, a molecular genetic diagnosis is crucial as it can predict the most appropriate treatment and genuinely improve quality of life [3]. The most striking example is seen for NDM patients with a mutation in the K_ATP_ channel genes, who can be treated effectively with oral sulfonylureas that directly bind the SUR1 regulatory subunit of the channel, rather than requiring life-long insulin therapy which usually provides poor glycemic contol [4], [5], [6].

Most developed countries offer DNA testing for NDM patients to establish a personal molecular genetic diagnosis for family counselling and to plan personalized pharmacotherapy. When severe hyperglycaemia is detected in a neonate, it is difficult to predict whether NDM will be transient or permanent. If the young patient does not have extrapancreatic features or a family history of diabetes (especially in a consanguineous context), it is suggested to first search for a chromosome 6q24 abnormality or for a *KCNJ11* mutation, as these NDM aetiologies are the most frequent, and then for mutations in *ABCC8* and *INS* if the first tests are negative [1], [3]. As *KCNJ11*, *ABCC8* and *INS* altogether represent 42 coding exons, sequencing these genes using the standard Sanger protocol is obviously tedious, long and costly. If this first set of gene sequencing is negative, further molecular analysis of the other NDM genes is generally not performed. This current approach to molecular diagnosis of NDM provides only a limited sequencing of the known NDM genes and no assessment of possible modifier genetic loci elsewhere in the genome: a more comprehensive cost efficient methodology to scrutinize every new NDM case is necessary.

In the present study, we demonstrate the feasibility of next-generation whole exome sequencing (WES) for the molecular diagnosis of a patient with NDM without any extrapancreatic features or family history of diabetes. Despite previous negative Sanger sequencing of *ABCC8* by a hospital laboratory, we identified a novel non-synonymous mutation in this gene through WES. We show that this cutting-edge novel technology is more comprehensive, less labour intensive and thus cheaper for NDM diagnosis than standard sequencing protocols.

## Results

The patient's clinical record reported that he has developed severe hyperglycemia, ketoacidosis and weight loss at two months of age. HLA typing showed neutral alleles for type 1 diabetes mellitus susceptibility. Pancreas ultrasound scan was normal and the patient did not show any specific extra-pancreatic clinical features. He was firstly treated with continuous subcutaneous insulin infusion during two years with rather low dose of insulin (<0.5 units/kg/day) for a pretty good metabolic control (A1C <8.5%). He was then switched with a basal-bolus scheme for technical adverse outcome. The patient is currently 20 years old. He is treated with 1.1 units/kg/day of insulin, with A1C values ranging between 8 and 9%. He always had an attention disorder and a learning disability without obvious motor symptoms or epilepsy.

After target enrichment, the whole exome DNA library from the patient was sequenced in 76 base-pairs (bp) paired-end reads, using two channels of the sequencing instrument. The WES generated 34,600,000 bp of nucleotide sequences mapping once to the targeted exome, which achieved a mean coverage of 122×of the target exome. WES identified 55,202 targeted DNA variants from the reference human genome sequence NCBI36/hg18, of which 4,463 were novel compared to the public database dbSNP130 and the eight HapMap exomes sequenced by Ng et al. [7] (Table 1). More specifically, we found 407 genes with one or more novel mutations including missense coding SNPs, gains of STOP codon and frameshift mutations (Table 1).

**Table 1:** Number of mutations identified through the WES analysis of DNA sample from the PNDM patient.

Sample		PNDM patient
Total targetted SNPs **(Novel** * **)**		55,202 **(4,463)**
Homozygous SNPs	Total SNPs **(Novel** * **)**	22,030 **(589)**
	Concordance* (%)	100
	Sensitivity† (%)	91.8
Heterozygous SNPs	Total SNPs (**Novel** *)	33,172 **(3,872)**
	Concordance† (%)	99.5
	Sensitivity‡ (%)	95.1
Synonymous coding SNPs	Homozygous **(Novel** * **)**	3,262 **(14)**
	Heterozygous **(Novel** * **)**	4,780 **(277)**
Missense coding SNPs (a)	Homozygous **(Novel** * **)**	2,907 **(18)**
	Heterozygous **(Novel** * **)**	4,264 **(430)**
Gains of STOP codon (b)	Homozygous **(Novel** * **)**	16 **(0)**
	Heterozygous **(Novel** * **)**	30 **(7)**
Insertions or deletions (c)	Homozygous **(Novel** * **; Novel frameshift)**	1,748 **(951; 0)**
	Heterozygous **(Novel** * **; Novel frameshift)**	409 **(284; 0)**
Genes with one or more (a),(b) or (c) mutations **(Novel** * **)**		4,495 **(407)**

*Novel: a novel mutation means that it is not present in the public database dbSNP130 and the eight HapMap exomes sequenced by Ng et al. [7].

† *Concordance*: % of similar allele assignment among exomic mutations detected on the Illumina Human1M-Duo array and those discovered by WES.

‡ *Sensitivity*: % of exomic mutations present on the Illumina Human1M-Duo array that have been discovered by WES.

To validate our WES data and analysis protocol, the patient's DNA was genotyped using an Illumina Human1M-Duo array, which contains 8,500 single nucleotide polymorphisms (SNPs) located in exons captured by the Agilent SureSelect technology. WES identified 7,969 exomic SNPs present on the genotyping array (93.8%), and showed high concordance rates for homozygous and heterozygous calls (100% and 99.5% and respectively). By using only one channel of the sequencing instrument, the generated sequence achieved a mean coverage of 65×of the target genome and 88.9% of the exomic SNPs present on the genotyping array were identified. These results would suggest that our WES based on the use of two channels of the genome analyzer has a low false negative rate for detecting exomic mutations, which is necessary to achieve accurate molecular diagnosis for patients presenting with monogenic disorders.

In 2004, single-stranded Sanger sequencing analysis of the patient's DNA by a hospital unit showed no evidence for coding mutations in *ABCC8*. Also, no abnormalities in *KCNJ11*, *INS* and chromosome 6q24 were reported by the molecular diagnostic laboratory of Robert Debré Hospital (Paris, France). Since these assessments were performed six years ago, we checked again for mutations in these three genes as well as in all other genes known to be involved in monogenic forms of diabetes. Unexpectedly, we identified a novel heterozygous non-synonymous mutation c.1455G>C/p.Q485H in the 9^th^ exon of *ABCC8*. Based on this startling result, we used a standard Sanger sequencing protocol, to study the patient's DNA sample available in the CNRS-UMR8199 unit (Lille, France) as well as the sample stored at the Robert Debré hospital (Paris, France). In both cases, we identified the p.Q485H mutation in *ABCC8*, confirming our WES results. In addition, retrospective re-examination of the data generated six years ago indicated that the p.Q485H mutation was present in the original *ABCC8* exon 9 sequences (chromogram). The *ABCC8* p.Q485H mutation was not found in 348 French nondiabetic individuals or in the patient's mother, father and young brother, all of whom are normoglycemic. The p.Q485H mutation affects an amino acid that is located in the transmembrane domain 1 (TMD1) of the ABCC8/SUR1 core; and that is highly conserved across species (Rhesus, Mouse, Dog, Rabbit, Elephant, Opossum, Platypus, Chicken, Lizard, Stickleback, X_Tropicalis, Tetraodon) according to the UCSC (NCBI/hg18) comparative genomics alignment pipeline (http://www.bx.psu.edu/miller_lab/). We evaluated the possible functional significance of the p.Q485H mutation by the PolyPhen-2 (Polymorphism Phenotyping v2) software which uses sequence- and structure-based criteria to predict the putative impact of point mutations on the structure and function of human proteins [8]: the p.Q485H mutation is predicted ‘probably damaging’ with a score of 0.999 (the score of 1 indicating the most damaging mutation). Following the identification of this *ABCC8* mutation in the patient's DNA, a switch from insulin to oral sulfonylurea treatment will be tried soon at Necker Hospital (Paris, France).

## Discussion

In the present study, we demonstrate for the first time that WES can be seen as a relevant alternative for molecular diagnosis of NDM. Since an accurate molecular diagnosis for this condition can lead to very dramatic improvements in patient care, development of reliable and cost efficient methods for quick and accurate DNA analysis are of major interest.

Currently, patients with NDM are evaluated using Sanger sequencing, which is far more expensive per sequenced base-pairs than WES. Indeed, the standard sequencing of a single PCR fragment costs 67.50€ ($82) for the only French hospital laboratory specialized in NDM molecular diagnosis (Robert Debré hospital, Paris). This price includes consumables, equipment amortization, personnel salary and hospital overhead costs. Therefore, the total cost for the French National Insurance for the sequencing of *KCNJ11*, *ABCC8* and *INS* alone, which requires 42 PCR fragments is 2,835€ ($3,440; ∼0.45€ or ∼$0.55 per bp). In comparison, the all inclusive cost of WES for NDM, which will detect mutations in *KCNJ11*, *ABCC8* and *INS* as well as rarer genetic aetiologies of NDM, is currently 3,274€ ($4,146; <<0.001€ or $ per bp) per sample, by performing a sequencing on two channels and in 76 bp paired-end configuration (CNRS-UMR8199, Lille, France). Moreover, it is very likely that WES cost will fall in next months towards 2,000€ ($2,528) or even less.

We believe that the WES protocol is less labour intensive and time-consuming than the standard Sanger protocol for genetically heterogeneous disorders requiring several large genes to be screened. A WES run involving four DNA samples can be completed in two weeks, including the time required to analyse the data, which is comparable to the time required by current Sanger sequencing of *ABCC8* only with its 39 exons. The p.Q485H mutation was missed six years ago by the research assistant in charge of the sequence reading. Although we can assume that mutation detection bio-informatics tools were less efficient a few years ago (the hospital laboratory used the PhredPhrap software in 2004) and that current methods are more accurate, the Sanger protocol and specially the semi-automated reading of sequence traces is always laborious and demanding (thus expensive), and a double-check of sequence readings by two different persons is performed in several French diagnostic laboratories in order to avoid any errors in the mutation identification process.

WES method is not only a cost-effective tool for molecular diagnosis; it should be also seen as an excellent tool for further genetic research and identification of novel causal mutations. Indeed, in the French NDM cohort, half of PNDM cases are still not elucidated [9], [10]. Classical linkage analyses are generally not successful as many NDM mutations occur *de novo* or are not fully penetrant. Most NDM genes have been found via candidate gene analyses but this approach has now reached its limits [11]. However, WES typically yields thousands of ‘novel’ genetic variants (*i.e.* not yet present in human genome variants databases). Therefore, the identification of truly causal variants would be strongly facilitated by the development of a high quality WES database of novel mutations found in both elucidated cases or in cases of unknown aetiology as well as in controls coming from same ethnicity. WES would also permit the identification of putative NDM modifier genes, a very challenging task for targeted gene analysis.

We are quite confident that the p.Q485H mutation is likely to be functional given the non ambiguous prediction of its putative damaging effect. In addition, the clinical data from the patient fit well with the features of PNDM linked to a *ABCC8* mutation (*e.g. de novo* mutation associated with very early-onset of the disease and attention disorder) [4], [12].

We believe that other NDM patients should be assessed with the same protocol as DNA quality may change the WES accuracy. Also, our DNA capture was not totally perfect as we could miss approximately 6% of exomic SNPs present in the high-resolution oligonucleotide genotyping array. Furthermore, the exomic coverage was not homogeneous between NDM genes (Table 2), thus we could suspect that the WES accuracy would not be the same for all NDM genes. Therefore, despite high WES mean coverage and elevated rates of both concordance and sensitivity in mutation detection, it is also necessary to assess and to verify the homogeneity of the target capture, specially in the genes of interest that have to be screened for molecular diagnosis (Table 2).

**Table 2:** Details on exomic sequencing depth in NDM genes, obtained through the WES of the PNDM patient.

NDM genes	Chromosome	Start	End	Number of coding exons	Exomic size (bp)	Number of mapped 76 bp exomic reads	Mean exomic coverage	% of sequenced exomic genes according to several depth thresholds			
								≥8×	≥20×	≥50×	≥100×
*KCNJ11*	chr11	17365042	17366214	1	1173	984	57.1×	100	88.7	50.5	13.6
*ABCC8*	chr11	17371114	17454899	39	4746	6235	90.5×	94.6	85.0	54.5	31.6
*INS*	chr11	2137658	2138777	2	333	97	24.4×	87.1	65.8	0	0
*GCK*	chr7	44164197	44159587	12	2012	1055	46.1×	70.8	44.1	19.0	14.4
*GLIS3*	chr9	3818272	4115864	9	2328	3672	130.4×	84.8	79.4	67.8	39.9
*EIF2AK3*	chr2	88638369	88707907	17	3351	9507	214.1×	92.2	92.0	87.1	81.4
*PDX1*	chr13	27392276	27396838	2	852	179	27.2×	40.3	24.5	3.9	0
*PTF1A*	chr10	23521466	23522840	2	986	405	59.9×	38.8	36.4	26.3	7.0
*SLC2A2*	chr3	172198386	172227153	11	1575	3669	170.9×	99.6	99.2	88.8	69.8
*HNF1B*	chr17	33121488	33178988	9	1674	2089	94.5×	93.3	83.4	53.5	34.5
*FOXP3*	chrX	48994739	49001906	11	1296	402	25.6×	69.7	51.9	13.8	0

Knowing that the capture technology is improving day after day (by enriching exomic loci poorly captured with the previous kits), our present study suggests that it will be possible to soon update the protocols for molecular diagnosis of NDM [3], [13]. We propose that after discovery of severe hyperglycemia in a neonate who is negative for serological markers of type 1 diabetes, a preliminary assessment of abnormalities of chromosome 6q24 can be performed (as at this stage, it is too early to guess NDM will be permanent or transient) followed by the search of a mutation in both *KCNJ11* and *INS* using Sanger sequencing as these two genes can be easily and quickly sequenced. If negative, we propose a WES analysis of the patient's DNA which is the most comprehensive way to fully explore the molecular causes of this NDM case.

## Materials and Methods

### Study participant and DNA samples

For WES, we selected a patient of European origin, diagnosed with PNDM who was referred to the French Network for the Study of Neonatal Diabetes Mellitus [9]. He was born from non consanguineous parents and had no intra-uterine growth retardation (birth weight 2,900 g/birth length 50 cm at 40 gestational weeks). He underwent a thorough clinical examination and his medical records were reviewed. Assessment of neurological outcome was also performed. Initial diagnostic testing for mutations in *KCNJ11*, *ABCC8* and *INS*, and for chromosome 6q24 abnormalities was negative. DNA samples from the patient's parents and his young brother were available for genetic testing.

### Ethics Statement

The study was approved by the local ethics committees (Assistance Publique – Hôpitaux de Paris, ClinicalTrials.gov Identifier: NCT00610038), and both parents gave written informed consent for the genetic testing of their child.

### Targeted capture and massive parallel sequencing

Approximately 187,000 coding exons from 3 µg of genomic DNA from the patient were captured using the Agilent SureSelect Human All Exon kit, following the manufacturer's protocols. Briefly, DNA was sheared by acoustic fragmentation (Covaris) and purified using the QIAquick PCR Purification Kit (Qiagen). The quality of the fragmentation and purification was assessed with the Agilent 2100 Bioanalyzer. The fragment ends were repaired and adaptors were ligated to the fragments (NEBNext DNA sample prep, New England Biolabs). The resulting DNA library was purified using the QIAquick PCR Purification Kit, amplified by PCR and captured by hybridization to the biotinylated RNA library “baits” (Agilent). Bound genomic DNA was purified with streptavidin coated magnetic Dynal beads (Invitrogen) and re-amplified. The whole-exome DNA library was sequenced on the Illumina Genome Analyzer IIx in 76 bp paired-end reads and using two channels.

### Read mapping, variant analysis and quality test of the sequencing protocol

Sequence reads were mapped to the reference human genome (UCSC NCBI36/hg18) using the ELANDv2 software (Illumina). Variant detection was performed with the CASAVA software (version 1.6, Illumina) and filtered to fit a CASAVA quality threshold ≥10 and depth of ≥8× CASAVA filters duplicate reads and reads without matched pairs.

A genomic DNA sample from the patient was genotyped on the Illumina Human 1M-Duo DNA Analysis BeadChips (with a call rate of 99.3%) as previously described [14]. We assessed the rate of exomic single nucleotide polymorphisms (SNPs) present on the array that were identified by WES and we calculated the concordance between the two methods.

### Mutation validation

The p.Q485H mutation identified via WES was confirmed using the Sanger method. Primer sequences and PCR conditions are available upon request to authors. The PCR fragment fitting the 9^th^ exon of *ABCC8* was sequenced using a standard protocol and the automated 3730xl DNA Analyser (Applied Biosystems). Electrophoregram reads were assembled and analysed with the Variant Reporter software (Applied Biosystems).
